# Fitness Trade-offs Result in the Illusion of Social Success

**DOI:** 10.1016/j.cub.2015.02.061

**Published:** 2015-04-20

**Authors:** Jason B. Wolf, Jennifer A. Howie, Katie Parkinson, Nicole Gruenheit, Diogo Melo, Daniel Rozen, Christopher R.L. Thompson

**Affiliations:** 1 Department of Biology and Biochemistry, University of Bath, Claverton Down, Bath BA2 7AY, UK; 2 Faculty of Life Sciences, Michael Smith Building, University of Manchester, Oxford Road, Manchester M13 9PT, UK; 3 Departamento de Genética e Biologia Evolutiva, Instituto de Biociências, Universidade de São Paulo, São Paulo, SP, Brazil; 4 Institute of Biology, Leiden University, Sylvius Laboratory, Sylviusweg 72, PO Box 9505, 2300 RA Leiden, the Netherlands

## Abstract

Cooperation is ubiquitous across the tree of life, from simple microbes to the complex social systems of animals [1]. Individuals cooperate by engaging in costly behaviors that can be exploited by other individuals who benefit by avoiding these associated costs. Thus, if successful exploitation of social partners during cooperative interactions increases relative fitness, then we expect selection to lead to the emergence of a single optimal winning strategy in which individuals maximize their gain from cooperation while minimizing their associated costs [2]. Such social “cheating” appears to be widespread in nature [3], including in several microbial systems [4–11], but despite the fitness advantages favoring social cheating, populations tend to harbor significant variation in social success rather than a single optimal winning strategy. Using the social amoeba *Dictyostelium discoideum*, we provide a possible explanation for the coexistence of such variation. We find that genotypes typically designated as “cheaters” [12] because they produce a disproportionate number of spores in chimeric fruiting bodies do not actually gain higher fitness as a result of this apparent advantage because they produce smaller, less viable spores than putative “losers.” As a consequence of this trade-off between spore number and viability, genotypes with different spore production strategies, which give the appearance of differential social success, ultimately have similar realized fitness. These findings highlight the limitations of using single fitness proxies in evolutionary studies and suggest that interpreting social trait variation in terms of strategies like cheating or cooperating may be misleading unless these behaviors are considered in the context of the true multidimensional nature of fitness.

## Highlights

• *D. discoideum* “cheater” genotypes produce smaller spores with reduced viability
• *D. discoideum* “losers” invest in larger spores with higher viability
• A number/viability trade-off means different social strategies have similar fitness
• Cheating may be illusory unless viewed in the context of multidimensional fitness

## Graphical Abstract

**Figure:**
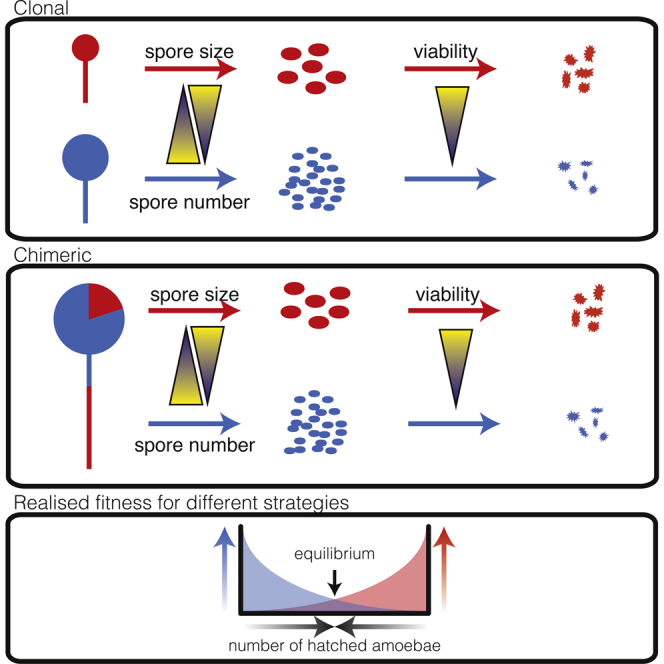

## Teaser

If exploitation of social partners increases fitness, then selection should favor one optimal winning strategy. However, cheaters and losers often coexist. Here, Wolf et al. show that different social strategies can coexist in the social amoeba *D. discoideum* due to trade-offs between social traits, meaning that simple views of cheating may be illusory.

## Results and Discussion

### Social Success in *D. discoideum*

*D. discoideum* live as single-celled amoebae in terrestrial habitats, but when their food is depleted, large numbers (∼10^5^) of individuals aggregate to form a multicellular fruiting body [13, 14]. The fruiting body is comprised of dead stalk cells that sacrifice themselves to hold aloft a ball of viable spores. Importantly, because fruiting bodies can contain a mixture of different genotypes, this is expected to lead to selection for exploitative social “cheaters,” which in *D. discoideum* have historically been defined simply as those strains that are overrepresented in the spore population of chimeric fruiting bodies [12, 15–20]. Consistent with earlier experiments [21, 22], we found that a set of genotypes isolated from a small geographic area in North Carolina [23] showed highly significant quantitative genetic variation (i.e., among-strain variation, *H*^2^) in the relative number of spores produced by each strain after amoebae were mixed in a 50:50 ratio and allowed to undergo chimeric development (*H*^2^ = 0.35, credible interval (CI) = [0.16, 0.62]; see Figure S1). This resulted in a linear (transitive) dominance hierarchy (*t*_tri_ = 0.73, p < 0.001; see [24]) with clear cheaters and “losers” when defined solely in terms of spore numbers. These observations thus raise a critical question: what processes maintain such variation in apparent social success in this species?

### Trade-offs Exist between Spore Size, Number, and Viability

One mechanism by which variation in social success could persist is if fitness gains during social competition are offset by inherent costs in another context (e.g., social traits expressed in a non-social context or through pleiotropic links between different social traits or social and non-social traits). Such trade-offs could potentially lead to the coexistence of diverse social behaviors, where different strategies have similar overall fitness, and hence the variation is nearly neutral and persists at mutation-selection balance [25]. It is also possible that the traits mediating social interactions are shaped primarily by selection in a non-social context, which incidentally gives rise to variation in social fitness, but only as a neutral byproduct.

Fitness trade-offs for non-social traits are known to be widespread [26]. For example, genotypes that produce greater numbers of offspring often compromise their investment into each individual offspring [27]. These quality-versus-quantity trade-offs (often stated in terms of a size/number trade-off) are ubiquitous in nature [28], with the optimum balance depending on the organism and the environment [29]. However, in the *D. discoideum* social system, where spores can be thought of as “offspring,” studies have used only the relative number of spores produced by different genotypes during social encounters as a measure of relative social success and thus social fitness, without consideration of the quality of those spores. Therefore, this interpretation relies on the implied and untested assumption that all offspring are created equal. Here we challenge this assumption, reasoning that *D. discoideum* genotypes could potentially produce large numbers of small, low-quality progeny (i.e., small spores with relatively low viability) or invest in smaller numbers of larger but higher-quality progeny (i.e., larger spores with higher viability). As the two strategies could result in the same overall fitness return, such a trade-off could result in the persistence of variation in spore investment strategies, which are in turn manifested as variation in social strategies when the relative numbers of spores produced in chimeras is considered as the sole measure of “success.”

**Figure 1:**
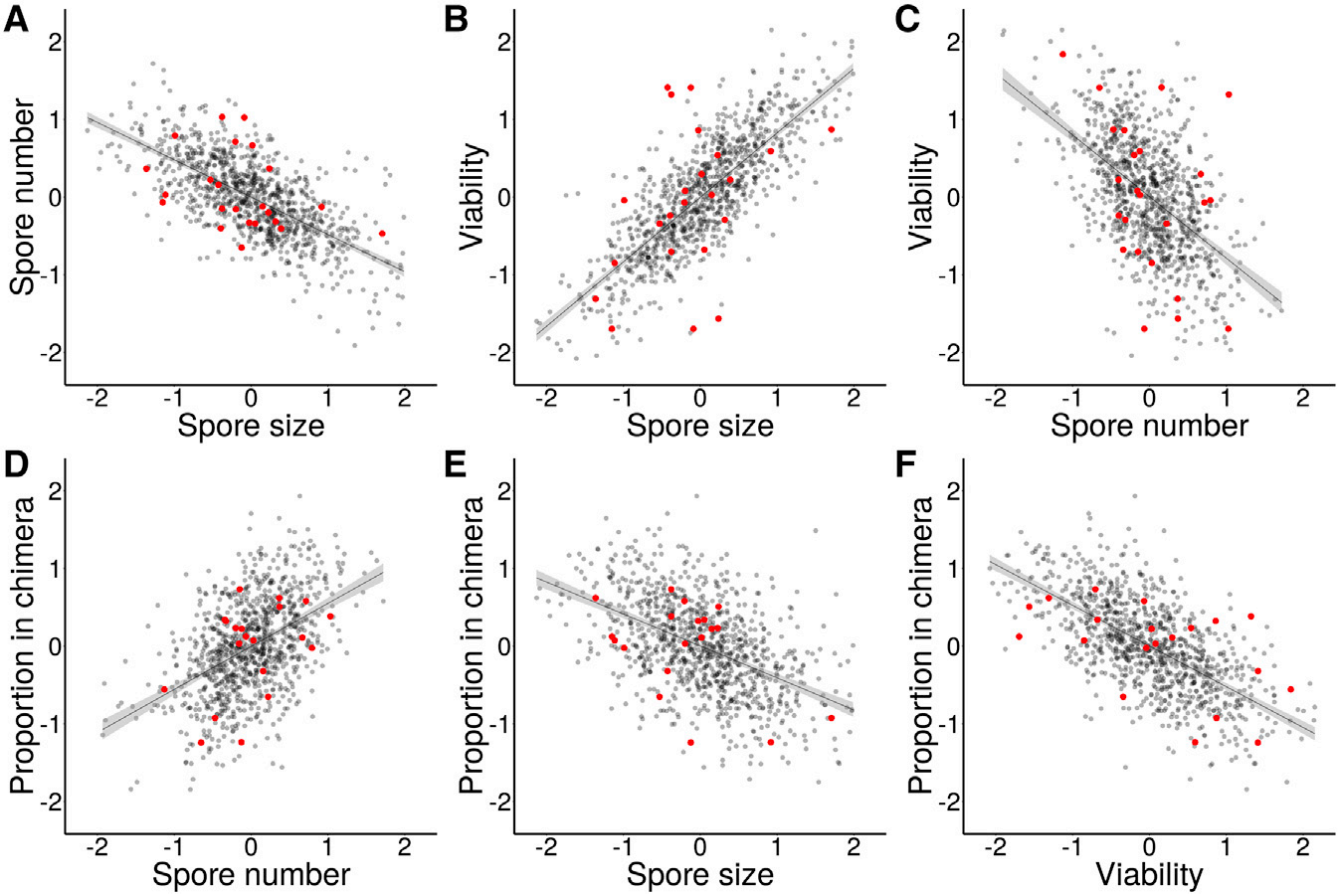
The Pairwise Genetic Relationships between the Four Fitness-Related Traits (A)–(C) show pairwise relationships between different non-social traits, while (D)–(F) show the relationship between these three non-social traits and the proportion of spores in a chimeric sporehead. All traits are illustrated in standard deviation units, with the x axis scaled the same way in all figures. The individual gray points are the simulated strains from the Bayesian model generated by MCMCglmm, and the red points are the genotypic means. The diagonal lines in the figures represent the best-fit line from linear regression, with the gray band surrounding each line illustrating the 95% confidence interval.

To investigate the hypothesis that non-social trade-offs might explain the persistence of variation in social traits by permitting the coexistence of diverse social strategies, we quantified the total number, size, and viability of spores produced by each strain. We identified significant quantitative genetic variation for the total number of spores produced (*H*^2^ = 0.25, CI = [0.12, 0.41]), spore size (*H*^2^ = 0.59, CI = [0.20, 1.12]), and spore viability (*H*^2^ = 0.62, CI = [0.19, 1.12]) (Figure S1). Moreover, we identified significant genetic correlations between all three measures (Figure 1). First, the total number of spores produced was found to be significantly negatively genetically correlated with spore size (*r* = −0.72, 95% credible interval, CI = [–0.95, –0.43]; Figure 1A), demonstrating that strains producing more spores do so at least in part by making smaller spores. Second, variation in spore size was significantly positively genetically correlated with differences in spore viability (*r* = 0.86, CI = [0.65, 0.99]; Figure 1B), indicating that larger spores hatch and survive better than smaller spores. Third, the variation in spore viability was significantly negatively genetically correlated with variation in the number of spores produced (*r* = −0.54, CI = [−0.88, −0.22]; Figure 1C), revealing that genotypes producing more, smaller spores also produce spores with reduced viability.

### Social Success Comes at the Cost of Decreased Spore Viability

Having identified significant variation in traits associated with apparent social success and spore traits, we next asked how these traits translate into the total realized social fitness of each genotype (where “social fitness” refers to the relative fitness of different genotypes resulting from social interactions). We found that the relative representation of spores of each genotype after chimeric development (chimeric representation) was positively genetically correlated with total number of spores produced (*r* = 0.50, CI = [0.13, 0.79]; Figure 1D) and negatively genetically correlated with spore size (*r* = −0.55, CI = [−0.85,−0.18]; Figure 1E), suggesting that genotypes that produce more spores consequently have higher representation in the chimeric sporehead but do so by producing more but smaller spores. However, because spore viability scales negatively with spore size, this leads to a negative genetic correlation between viability and chimeric representation (*r* = −0.69, CI = [−0.95, −0.40]; Figure 1F). Together, these results lead to the conclusion that genotypes that achieve higher representation of spores in chimeric fruiting bodies do so by producing greater numbers of lower-viability spores.

### Trade-offs Negate Fitness Gained through Sporehead Representation

**Figure 2:**
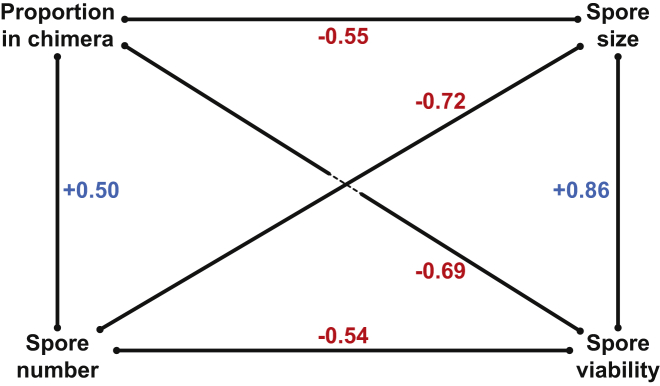
Summary of the Genetic Correlational Structure of the Four Traits The four traits are connected by the six genetic correlations, which were estimated by MCMCglmm [30]. Positive correlations appear in blue, and negative correlations appear in red. All correlations are significant (credible intervals are given in the text).

**Figure 3:**
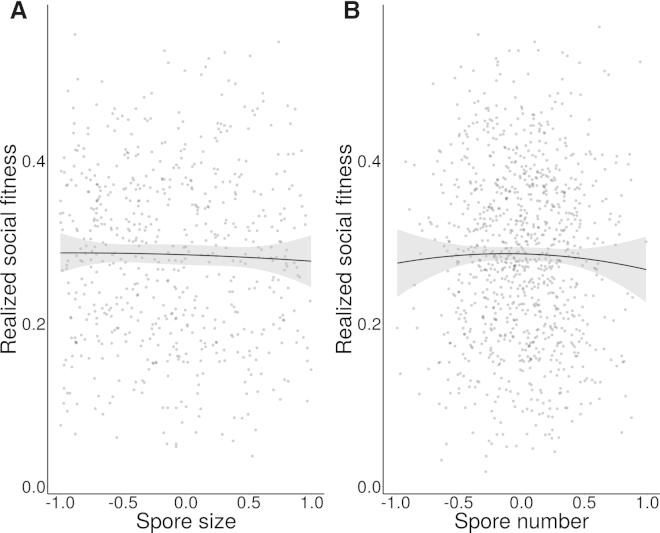
Relationship between Realized Social Fitness and Spore Size and Number The relationships between realized social fitness (modeled as the product of proportional representation in the chimeric sporehead and spore viability) and spore number (A) and spore size (B) are illustrated using the simulated strains from the Bayesian model (gray points) with a quadratic regression curve (black line) and 95% confidence interval (gray band).

By accounting for these correlations between traits (summarized in Figure 2), we estimated a realized social fitness value that discounts representation of spores during chimeric development by the subsequent viability of the spores produced. This analysis clearly demonstrates that, due to trade-offs between traits, the relationship between spore size (Figure 3A) or spore number (Figure 3B) and realized social fitness is essentially flat. Therefore, despite significant variation in both of these underlying traits, which ultimately determine components of fitness, this variation appears to be effectively neutral in terms of realized social fitness.

### Trade-offs Help Explain the Coexistence of Cheaters and Losers

Social systems and measurements of social success are often viewed from the perspective of a single fitness-related trait (e.g., [12, 22]), which is then used as a proxy for total fitness. Although this narrow consideration is sometimes unavoidable given the challenge of measuring overall fitness in a relevant environmental context, our results reveal that this narrow perspective may produce misleading conclusions because it ignores the fact that organisms are inherently “multidimensional,” being composed of suites of traits that together determine their fitness. Realized fitness of any genotype will therefore be the product of different, potentially conflicting components. Moreover, examining fitness through this multidimensional lens highlights the fact that traits affecting different aspects of life history not clearly associated with social interactions could have indirect effects on social success [31]. As a consequence, although each individual trait may appear to confer a fitness advantage (and therefore be under directional selection), the multidimensional system of traits is constrained by trade-offs, resulting in no net selection on the set of traits when viewed as a whole [32].

The label of “cheater” has often been applied to *D. discoideum* genotypes that have a higher representation of spores than some of their competitors during chimeric fruiting body development. One way this could occur is if genotypes exhibit differences in developmental signaling that lead to different ratios of spore or stalk cells [33]. Although this mechanism is possible, it is hard to envisage how it could lead to differences in total spore number, as well as affecting the size or viability of resulting spores. We therefore believe it is more likely that both trade-offs arise from differences in the number of reductive cell divisions that occur during the multicellular stages of the life cycle. Indeed, there is widespread evidence supporting the idea that some cells, and especially those destined to become spores, do indeed undergo division during the migratory slug phase [34, 35]. If resources and biomass were limiting and unequally partitioned in the multicellular slug, such reductive division would result in smaller cells, thus providing a plausible explanation for the resulting smaller spores observed. This latter pattern appears to explain much of the variation observed, given that different spore production strategies appear to result in similar social fitness as a result of trade-offs. Under this scenario, different spore production strategies are nearly neutral in terms of their influence on social fitness (Figure 3), and hence the continuum of social behavioral strategies seen in these genotypes may simply reflect low selection pressure on social traits.

It is important to note, however, that although we have shown that relative representation in the sporehead is a poor measure of true social success, when interactions are viewed from the perspective of realized social fitness (which includes both spore number and viability) we find that there remains variation in social fitness that should reflect the true nature of cheaters and losers in this system (Figure 3). Similarly, in other microbial systems such as *Myxococcus* and *Pseudomonas*, cheater genotypes that exploitatively outcompete cooperators in mixed groups have been described when social fitness is measured in terms of the relative production of viable spores or cells, respectively [5, 10]. This is almost certainly due to the fact that microbes have complex life cycles and live in heterogeneously structured environments where diverse intra- and interspecific dynamics will interact to affect fitness. Other life history traits that we have not examined are no doubt manifest in these ecologically relevant scenarios, and these in turn may directly or indirectly influence the coexistence of apparent social traits [36].

Our study therefore has clear implications for understanding the evolution of social traits in terms of cheater or cooperator strategies. Most notably, our results illustrate the importance of considering life history trade-offs when assessing social fitness: although many social systems, such as *D. discoideum*, may appear unbalanced with individuals that appear to “win,” these individuals are really no better off in terms of fitness than individuals that appear to “lose.” These observations may thus explain the paradoxical coexistence of substantial genetic variation in apparent social success in this and potentially other social organisms.

## Author Contributions

C.R.L.T., D.R., and J.B.W. conceived and designed the project and wrote the manuscript. J.A.H. performed the measurements of social success and spore numbers. K.P. and N.G. performed the measurements of spore size and viability. D.M. and J.B.W. designed the data analyses. D.M. performed all analyses.

## Supplemental Information

Document S1. Figure S1, Table S1, and Supplemental Experimental Procedures

Document S2. Article plus Supplemental Information
