# Supplementary material for: Fitness Trade-offs Result in the Illusion of Social Success
Source: Curr Biol. 2015 Apr 20;25(8):1086–90. doi: 10.1016/j.cub.2015.02.061 (PMC4406944; doi:10.1016/j.cub.2015.02.061)
Supplement: Document S1. Figure S1, Table S1, and Supplemental Experimental Procedures [file mmc1.pdf]

Current Biology
Supplemental Information

Fitness Trade-offs Result in the Illusion of Social Success

Jason B. Wolf, Jennifer A. Howie, Katie Parkinson, Nicole Gruenheit, Diogo Melo, Daniel Rozen, and Christopher R.L. Thompson

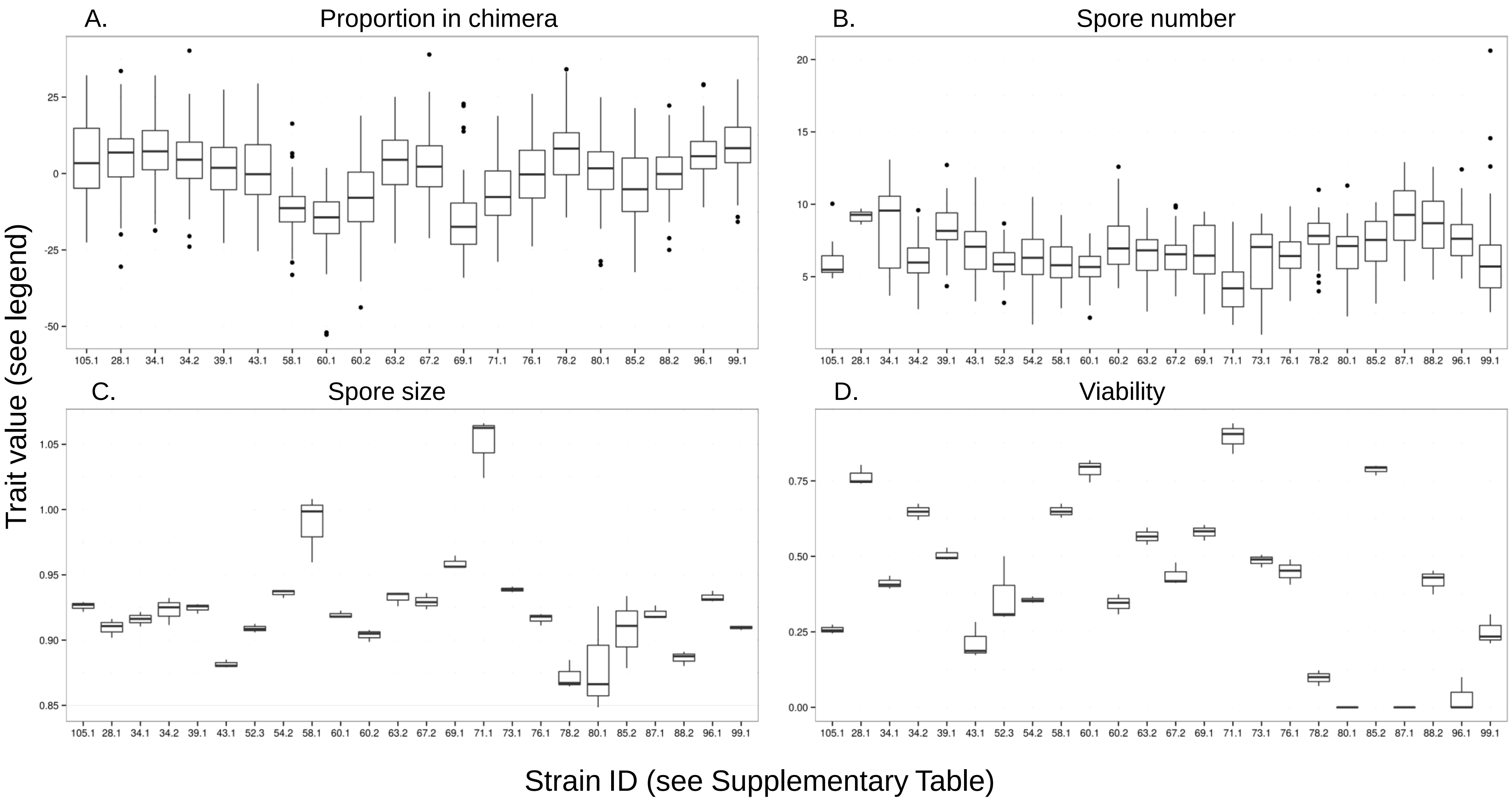

A.
Proportion in chimera
B.
Spore number
C.
Spore size
D.
Viability
Trait value (see legend)
Strain ID (see Supplementary Table)

Supplementary Figure 1

The distribution of the four traits for each strain. For each trait, strains are illustrated using a standard box plot, where the box extends from the first to the third quartiles (the 25th and 75th percentiles) with a line within the box indicating the location of the median. The upper and lower lines (whiskers) extend from the box to the highest and lowest values that are within 1.5 times the inter-quartile range (i.e., distance between the first and third quartiles). Data beyond the end of the whiskers are outliers and appear as individual points. A. The distribution of proportional representation in chimeric fruiting bodies. Values shown have been corrected for the effect of labelling and the social partner (to account for unbalanced combinations). The values are scaled such that they show the deviation expected from 50:50, where a value of zero means that the expected representation in chimeric sporeheads is 50%, while positive values show the expected degree of overrepresentation and negative values show the expected degree of underrepresentation. For example, a value of 25 indicates that the strain has an average representation in chimeric sporeheads of 75%. B. The number of spores in a clonal sporehead in units of 10^5 spores (e.g., a value of 8 would indicate 8 x 10^5 spores). The values shown have been adjusted to account for experimental block effects. C. The size of spores. Values plotted are the $\log_{10}$ of the raw values in μm, which is the scale used in the analyses (see Methods for more details). D. The viability of spores as a percentage. Values plotted are the squaren root of the raw values, which is the scale used in the analyses (see Methods for more details). For relationships between these data see Figure 1.

Supplementary Table 1

Strains (genotypes) used in all studies. All strains were originally isolated from Little Butt's Gap, North Carolina, USA [1] and obtained from the Dicty Stock Center. Strains listed in red were not used in the assays for social success. For trait data associated with these strains, see Figure 1.

NC28.1	NC43.1	NC60.1	NC69.1	NC78.2	NC88.2
NC34.1	NC52.3	NC60.2	NC71.1	NC80.1	NC96.1
NC34.2	NC54.2	NC63.2	NC73.1	NC85.2	NC99.1
NC39.1	NC58.1	NC67.2	NC76.1	NC87.1	NC105.1

EXPERIMENTAL PROCEDURES

Growth and maintenance of genotypes

Naturally occurring genotypes of *D. discoideum* used throughout the study were isolated from the same geographic region of Little Butt's Gap, North Carolina [1]. All traits were measured on a set of 24 strains (see Supplementary Table 1 for a list of strain IDs), except social success, which was measured for a subset of 20 strains. Genotypes were acquired from the Dicty Stock Centre and subsequently stored as frozen stocks. For experiments, stocks were grown on Schaeffer's sporulation medium (SM) agar plates in association with *Klebsiella aerogenes* (Ka) bacteria. For amoeba growth, 'clearing plates' containing 5 x 10^5 cells were plated with Ka and incubated at 22°C for approximately 36 hours. Growing cells were harvested before they began to aggregate and bacteria were removed by repeated washing and differential centrifugation in KK2 (16.1 mM KH_2PO_4, 3.7mM K_2HPO_4).

Estimating social success in chimera

Growing cells of each genotype were harvested, and re-suspended at 1 x 10^7 cells/ml. For fluorescent labelling, cells were shaken for 30 minutes with 10mM CMFDA Cell Tracker Green dissolved in DMSO; control cells were shaken with DMSO. For development, cells were plated on 1.5% KK2 L28 purified agar plates at a final density of 1.6 x 10^6 cells per cm^2. Different genotypes were mixed in a 50:50 ratio. The social success of each genotype was estimated by counting the percentage of fluorescent and non-fluorescent spores using a CyAn flow cytometer. Estimates of social success were made using all possible reciprocal pair-wise combinations of 20 genotypes. Each of the 380 pair-wise combinations was replicated an average of 5.16 times, resulting in a total sample size of 1960 measures of social success. Social success estimates were averaged across social partners and corrected for the labelling effect, using a mixed modelling approach. We modelled the fixed effect of Cell Tracker labelling and the random effect of the partner genotype on the proportion of the genotype in a chimeric fruiting body using the Mixed Procedure in SAS version 9.4 (SAS Institute, Cary, NC, USA), fitted by restricted maximum likelihood (REML). The residuals from this model were used for subsequent analyses.

To determine the degree of linearity (transitivity) in the pair-wise social success measures, we used the network measure 'triangle transitivity (t_{tri})' developed in Shizuka and McDonald [2], which tests the proportion of triads that are transitive relative to those that are non-transitive, scaled to the null expectation. The results are essentially identical using other

standard measures such as Kendall's *K* and Landau's *h* [see 2 for further details], so only a single measure is reported.

Spore size and viability

For estimates of spore size, strains were hatched onto SM agar with Ka bacteria and spores harvested from the resulting fruiting bodies into spore buffer (20mM EDTA and 0.1% NP-40). Spores were imaged at 40x magnification. Automated measurements of spore size (length, width, and total area) were made from three images per plate using ImageJ software [3]. Spores of each genotype were measured in three independent replicates, with at least 77 spores measured from each strain in each block (with a mean of 228.7 spores per strain per block for a total of 16,464 spores measured overall). Length, width and total area measures of spore size are highly intercorrelated, so we used spore length as the best estimate of spore size because it has the highest repeatability (in terms of the proportion of variance among strains). We used the replicated measures within each block to generate three completely independent estimates of spore size for each strain (i.e., one measure per strain per block). Because spore size is not normally distributed, we used the median spore size in each block as the best measure of spore size (almost identical results are achieved using the mean spore size in each replicate). The median spore sizes were then $\log_{10}$ transformed to achieve a normally distributed measure of spore size. This measure of spore size has a strain level repeatability of 71%. To measure spore viability, spores were harvested into spore buffer, counted using a haemocytometer and diluted to a density of 2×10^3 cells/ml. 200 spores were plated on SM agar with Ka bacteria with three replicates per strain. The number of visible clear plaques was counted after 4 days growth at 22 deg. To measure spore size and viability after chimeric development in 50:50 mixes, one genotype was labelled with CMFDA Cell Tracker as described above. Fluorescent and non-fluorescent spores were separated using a FACS Aria cell sorter, before size and viability were determined using the above methods. We found significant correlations between chimeric and clonal spore size (r = 0.94, p = 0.008) and spore viability (r = 0.84, p = 0.039), in a subset strains tested in pair-wise mixes. Therefore, clonal trait measures provide good estimates of chimeric traits, allowing us to estimate socially relevant traits through clonal development.

Spore number

To estimate total clonal spore counts, all fruiting bodies were washed from a plate containing clonal fruiting bodies into a known volume of spore buffer and the total number of spores

was estimated by direct haemocytometer counts. Each genotype was measured in at least 8 independent replicates, with an average of 33.6 replicates per strain. To account for differences across experimental blocks, we modelled the random effect of block on the spore count using the Mixed Procedure in SAS version 9.4 (SAS Institute, Cary, NC, USA), fitted by restricted maximum likelihood (REML). The residuals from this model were used for subsequent analyses.

Quantitative genetic analyses

Proportions of among strain variation in traits, which can be interpreted as either repeatabilities of a trait at the strain level of as broad-sense heritabilities (H^2), were estimated from the proportion of variance attributed to the random effect of genotype in a mixed model, fitted using restricted maximum likelihood (REML) in the Mixed Procedure in SAS version 9.4 (SAS Institute, Cary, NC, USA). Significance was estimated using a likelihood ratio test.

To estimate the genetic correlation between traits we first standardized each trait to a mean of zero and a variance of one. The four traits were used in a multivariate model fitted using MCMCglmm [4] using a Bayesian modification of the framework described by Fry [5] to estimate genetic variances of and genetic correlations among the four traits measured in the set of genotypes. In this model, the four traits are treated as measures of the same underlying trait and the genotype is used as the unit of repeated measurements. The model estimates the correlations for the traits at the level of genotypes, which represent genetic correlations since the traits were measured independently [see 5 for further details]. We used weakly informative independent Gaussian priors for the residual and mixed effect (genetic) variances. Model convergence was accessed by inspection of variable traces. The genetic correlations between traits were estimated as the mean of the posterior distribution. This estimate was similar to results from a maximum likelihood fitted model using SAS or lme4, with the added advantage that the posterior distribution of covariances can be used to create high probability confidence intervals (CI) for all heritabilities and genetic correlations, taking into account all sources of uncertainty in the system and allowing a straight forward test for significant difference from zero for all correlations [6]. To visualize the relationships between traits we used the posterior sampled covariance matrices and means to create a data set of simulated strains by drawing values from normal distributions in the scaled trait space. These values were plotted along with means for each observed strain to demonstrate the agreement between data and posterior simulations. These visualizations from the simulations also

illustrate the uncertainty for each of the relations between traits, and again their distribution takes into account every source of underlying uncertainty.

Modelling fitness

To understand the nature of selection on traits we first estimated the means and variances of the unscaled traits using MCMCglmm and, along with the correlations estimated for the scaled traits, used them to generate posterior simulations of strains in the unscaled space. We calculated expected 'social fitness' as the product of social success and spore viability (so relative representation in the clonal fruiting body discounted by the viability of the spores produced). To calculate fitness in each of the simulated strains we first transformed viability (which was estimated using the residuals from the mixed model) to the [0, 1] interval via an inverse logit transformation and scaled success to the same interval (so social success represents the average relative representation in the sporehead for a given genotype). Social fitness, as the product between viability and success, is then restricted to values between zero and one.

To understand the relationship between the underlying life-history traits (spore number and spore size) and components of fitness, we estimated the linear relationship between these traits and each of the fitness components (social success and viability). The net relationship between these traits and total fitness (i.e., the overall shape of the selection surface) was visualized using a quadratic regression curve.

References

S1. Francis, D., and Eisenberg, R. (1993). Genetic structure of a natural population of Dictyostelium discoideum, a cellular slime mold. Mol. Ecol. *2*, 385-392.

S2. Shizuka, D., and McDonald, D.B. (2012). A social network perspective on measurements of dominance hierarchies. Animal Behaviour *83*, 925-934.

S3. Schneider, C.A., Rasband, W.S., and Eliceiri, K.W. (2012). NIH Image to ImageJ: 25 years of image analysis. Nature Methods *9*, 671-675.

S4. Hadfield, J.D. (2010). MCMC Methods for Multi-Response Generalized Linear Mixed Models: The MCMCglmm R Package. Journal of Statistical Software *33*, 1-22.

S5. Fry, J.D. (2004). Estimation of genetic variances and covariances by restricted maximum likelihood using Proc Mixed. In Genetic analysis of complex traits using SAS, A.M. Saxton, ed. (Cary, N.C.: SAS Institute), pp. 11-34.

S6. Gelman, A., Carlin, J.B., Stern, H.S., Vehtari, A., and Rubin, D.B. (2013). Bayesian Data Analysis, Third Edition, (Chapman & Hall).
