# Supplementary material for: Fitness Trade-offs Result in the Illusion of Social Success
Source: Curr Biol. 2015 Apr 20;25(8):1086–90. doi: 10.1016/j.cub.2015.02.061 (PMC4406944; doi:10.1016/j.cub.2015.02.061)
Supplement: Document S2. Article plus Supplemental Information [file mmc2.pdf]

Report

Current Biology

Fitness Trade-offs Result in the Illusion of Social Success

Graphical Abstract

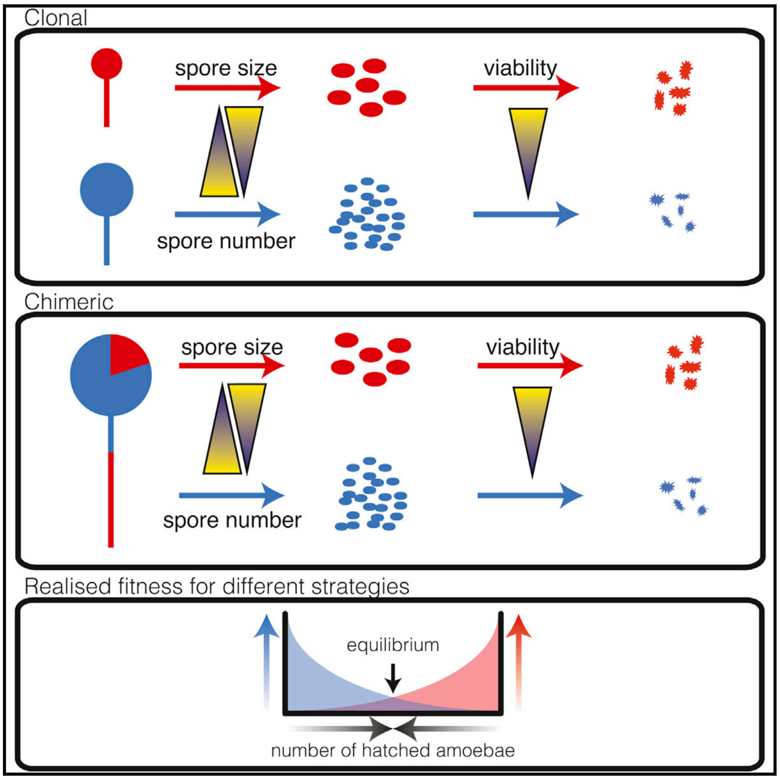

Authors

Jason B. Wolf, Jennifer A. Howie, ...,
Daniel Rozen,
Christopher R.L. Thompson

Correspondence

jason@evolutionarygenetics.org (J.B.W.),
christopher.thompson@manchester.ac.uk (C.R.L.T.)

In Brief

If exploitation of social partners increases fitness, then selection should favor one optimal winning strategy. However, cheaters and losers often coexist. Here, Wolf et al. show that different social strategies can coexist in the social amoeba *D. discoideum* due to trade-offs between social traits, meaning that simple views of cheating may be illusory.

Highlights

- *D. discoideum* "cheater" genotypes produce smaller spores with reduced viability
- *D. discoideum* "losers" invest in larger spores with higher viability
- A number/viability trade-off means different social strategies have similar fitness
- Cheating may be illusory unless viewed in the context of multidimensional fitness

Wolf et al., 2015, Current Biology *25*, 1086–1090
April 20, 2015 ©2015 The Authors
http://dx.doi.org/10.1016/j.cub.2015.02.061

CellPress

Current Biology
Report

Fitness Trade-offs Result in the Illusion of Social Success

Jason B. Wolf,[1,*] Jennifer A. Howie,[2] Katie Parkinson,[2] Nicole Gruenheit,[2] Diogo Melo,[3] Daniel Rozen,[4] and Christopher R.L. Thompson[2,*]
[1]Department of Biology and Biochemistry, University of Bath, Claverton Down, Bath BA2 7AY, UK
[2]Faculty of Life Sciences, Michael Smith Building, University of Manchester, Oxford Road, Manchester M13 9PT, UK
[3]Departamento de Genética e Biologia Evolutiva, Instituto de Biociências, Universidade de São Paulo, São Paulo, SP, Brazil
[4]Institute of Biology, Leiden University, Sylvius Laboratory, Sylviusweg 72, PO Box 9505, 2300 RA Leiden, the Netherlands
*Correspondence: jason@evolutionarygenetics.org (J.B.W.), christopher.thompson@manchester.ac.uk (C.R.L.T.)
http://dx.doi.org/10.1016/j.cub.2015.02.061
This is an open access article under the CC BY license (http://creativecommons.org/licenses/by/4.0/).

SUMMARY

Cooperation is ubiquitous across the tree of life, from simple microbes to the complex social systems of animals [1]. Individuals cooperate by engaging in costly behaviors that can be exploited by other individuals who benefit by avoiding these associated costs. Thus, if successful exploitation of social partners during cooperative interactions increases relative fitness, then we expect selection to lead to the emergence of a single optimal winning strategy in which individuals maximize their gain from cooperation while minimizing their associated costs [2]. Such social "cheating" appears to be widespread in nature [3], including in several microbial systems [4–11], but despite the fitness advantages favoring social cheating, populations tend to harbor significant variation in social success rather than a single optimal winning strategy. Using the social amoeba *Dictyostelium discoideum*, we provide a possible explanation for the coexistence of such variation. We find that genotypes typically designated as "cheaters" [12] because they produce a disproportionate number of spores in chimeric fruiting bodies do not actually gain higher fitness as a result of this apparent advantage because they produce smaller, less viable spores than putative "losers." As a consequence of this trade-off between spore number and viability, genotypes with different spore production strategies, which give the appearance of differential social success, ultimately have similar realized fitness. These findings highlight the limitations of using single fitness proxies in evolutionary studies and suggest that interpreting social trait variation in terms of strategies like cheating or cooperating may be misleading unless these behaviors are considered in the context of the true multidimensional nature of fitness.

RESULTS AND DISCUSSION

Social Success in *D. discoideum*

D. discoideum live as single-celled amoebae in terrestrial habitats, but when their food is depleted, large numbers (~10^5) of individuals aggregate to form a multicellular fruiting body [13, 14]. The fruiting body is comprised of dead stalk cells that sacrifice themselves to hold aloft a ball of viable spores. Importantly, because fruiting bodies can contain a mixture of different genotypes, this is expected to lead to selection for exploitative social "cheaters," which in *D. discoideum* have historically been defined simply as those strains that are overrepresented in the spore population of chimeric fruiting bodies [12, 15–20]. Consistent with earlier experiments [21, 22], we found that a set of genotypes isolated from a small geographic area in North Carolina [23] showed highly significant quantitative genetic variation (i.e., among-strain variation, H^2) in the relative number of spores produced by each strain after amoebae were mixed in a 50:50 ratio and allowed to undergo chimeric development (H^2 = 0.35, credible interval (CI) = [0.16, 0.62]; see Figure S1). This resulted in a linear (transitive) dominance hierarchy (t_{tri} = 0.73, $p < 0.001$; see [24]) with clear cheaters and "losers" when defined solely in terms of spore numbers. These observations thus raise a critical question: what processes maintain such variation in apparent social success in this species?

Trade-offs Exist between Spore Size, Number, and Viability

One mechanism by which variation in social success could persist is if fitness gains during social competition are offset by inherent costs in another context (e.g., social traits expressed in a non-social context or through pleiotropic links between different social traits or social and non-social traits). Such trade-offs could potentially lead to the coexistence of diverse social behaviors, where different strategies have similar overall fitness, and hence the variation is nearly neutral and persists at mutation-selection balance [25]. It is also possible that the traits mediating social interactions are shaped primarily by selection in a non-social context, which incidentally gives rise to variation in social fitness, but only as a neutral byproduct.

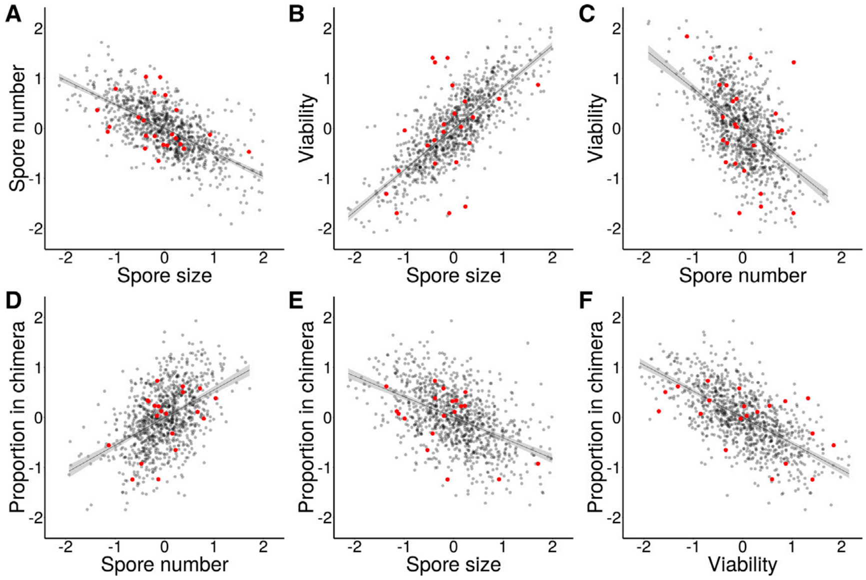

Figure 1. The Pairwise Genetic Relationships between the Four Fitness-Related Traits
(A)–(C) show pairwise relationships between different non-social traits, while (D)–(F) show the relationship between these three non-social traits and the proportion of spores in a chimeric sporehead. All traits are illustrated in standard deviation units, with the x axis scaled the same way in all figures. The individual gray points are the simulated strains from the Bayesian model generated by MCMCglmm, and the red points are the genotypic means. The diagonal lines in the figures represent the best-fit line from linear regression, with the gray band surrounding each line illustrating the 95% confidence interval.

Fitness trade-offs for non-social traits are known to be widespread [26]. For example, genotypes that produce greater numbers of offspring often compromise their investment into each individual offspring [27]. These quality-versus-quantity trade-offs (often stated in terms of a size/number trade-off) are ubiquitous in nature [28], with the optimum balance depending on the organism and the environment [29]. However, in the *D. discoideum* social system, where spores can be thought of as "offspring," studies have used only the relative number of spores produced by different genotypes during social encounters as a measure of relative social success and thus social fitness, without consideration of the quality of those spores. Therefore, this interpretation relies on the implied and untested assumption that all offspring are created equal. Here we challenge this assumption, reasoning that *D. discoideum* genotypes could potentially produce large numbers of small, low-quality progeny (i.e., small spores with relatively low viability) or invest in smaller numbers of larger but higher-quality progeny (i.e., larger spores with higher viability). As the two strategies could result in the same overall fitness return, such a trade-off could result in the persistence of variation in spore investment strategies, which are in turn manifested as variation in social strategies when the relative numbers of spores produced in chimeras is considered as the sole measure of "success."

To investigate the hypothesis that non-social trade-offs might explain the persistence of variation in social traits by permitting the coexistence of diverse social strategies, we quantified the total number, size, and viability of spores produced by each strain. We identified significant quantitative genetic variation for the total number of spores produced (H^2 = 0.25, CI = [0.12, 0.41]), spore size (H^2 = 0.59, CI = [0.20, 1.12]), and spore viability (H^2 = 0.62, CI = [0.19, 1.12]) (Figure S1). Moreover, we identified significant genetic correlations between all three measures (Figure 1). First, the total number of spores produced was found to be significantly negatively genetically correlated with spore size (r = −0.72, 95% credible interval, CI = [–0.95, –0.43]; Figure 1A), demonstrating that strains producing more spores do so at least in part by making smaller spores. Second, variation in spore size was significantly positively genetically correlated with differences in spore viability (r = 0.86, CI = [0.65, 0.99]; Figure 1B), indicating that larger spores hatch and survive better than smaller spores. Third, the variation in spore viability was significantly negatively genetically correlated with variation in the number of spores produced (r = −0.54, CI = [−0.88, −0.22]; Figure 1C), revealing that genotypes producing more, smaller spores also produce spores with reduced viability.

Social Success Comes at the Cost of Decreased Spore Viability

Having identified significant variation in traits associated with apparent social success and spore traits, we next asked how these traits translate into the total realized social fitness of each genotype (where "social fitness" refers to the relative fitness of different genotypes resulting from social interactions). We found that the relative representation of spores of each genotype after chimeric development (chimeric representation) was positively genetically correlated with total number of spores produced (r = 0.50, CI = [0.13, 0.79]; Figure 1D) and negatively genetically correlated with spore size (r = −0.55, CI = [−0.85,−0.18]; Figure 1E), suggesting that genotypes that produce more spores consequently have higher representation in the chimeric sporehead but do so by producing more but smaller spores. However, because spore viability scales negatively with spore size, this leads to a negative genetic correlation between viability and chimeric representation (r = −0.69, CI = [−0.95, −0.40]; Figure 1F). Together, these results lead to the conclusion that genotypes that achieve higher representation of spores in chimeric fruiting bodies do so by producing greater numbers of lower-viability spores.

Trade-offs Negate Fitness Gained through Sporehead Representation

By accounting for these correlations between traits (summarized in Figure 2), we estimated a realized social fitness value that discounts representation of spores during chimeric development

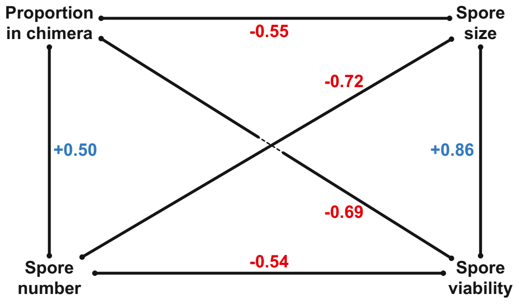

Figure 2. Summary of the Genetic Correlational Structure of the Four Traits

The four traits are connected by the six genetic correlations, which were estimated by MCMCglmm [30]. Positive correlations appear in blue, and negative correlations appear in red. All correlations are significant (credible intervals are given in the text).

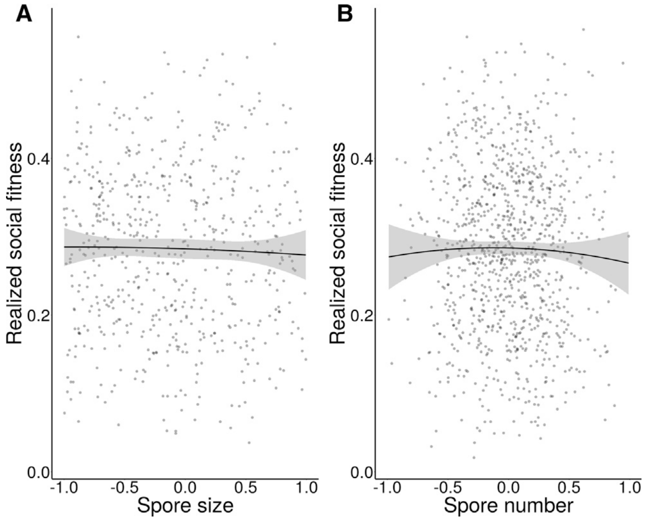

Figure 3. Relationship between Realized Social Fitness and Spore Size and Number

The relationships between realized social fitness (modeled as the product of proportional representation in the chimeric sporehead and spore viability) and spore number (A) and spore size (B) are illustrated using the simulated strains from the Bayesian model (gray points) with a quadratic regression curve (black line) and 95% confidence interval (gray band).

by the subsequent viability of the spores produced. This analysis clearly demonstrates that, due to trade-offs between traits, the relationship between spore size (Figure 3A) or spore number (Figure 3B) and realized social fitness is essentially flat. Therefore, despite significant variation in both of these underlying traits, which ultimately determine components of fitness, this variation appears to be effectively neutral in terms of realized social fitness.

Trade-offs Help Explain the Coexistence of Cheaters and Losers

Social systems and measurements of social success are often viewed from the perspective of a single fitness-related trait (e.g., [12, 22]), which is then used as a proxy for total fitness. Although this narrow consideration is sometimes unavoidable given the challenge of measuring overall fitness in a relevant environmental context, our results reveal that this narrow perspective may produce misleading conclusions because it ignores the fact that organisms are inherently "multidimensional," being composed of suites of traits that together determine their fitness. Realized fitness of any genotype will therefore be the product of different, potentially conflicting components. Moreover, examining fitness through this multidimensional lens highlights the fact that traits affecting different aspects of life history not clearly associated with social interactions could have indirect effects on social success [31]. As a consequence, although each individual trait may appear to confer a fitness advantage (and therefore be under directional selection), the multidimensional system of traits is constrained by trade-offs, resulting in no net selection on the set of traits when viewed as a whole [32].

The label of "cheater" has often been applied to *D. discoideum* genotypes that have a higher representation of spores than some of their competitors during chimeric fruiting body development. One way this could occur is if genotypes exhibit differences in developmental signaling that lead to different ratios of spore or stalk cells [33]. Although this mechanism is possible, it is hard to envisage how it could lead to differences in total spore number, as well as affecting the size or viability of resulting spores. We therefore believe it is more likely that both trade-offs arise from differences in the number of reductive cell divisions that occur during the multicellular stages of the life cycle. Indeed, there is widespread evidence supporting the idea that some cells, and especially those destined to become spores, do indeed undergo division during the migratory slug phase [34, 35]. If resources and biomass were limiting and unequally partitioned in the multicellular slug, such reductive division would result in smaller cells, thus providing a plausible explanation for the resulting smaller spores observed. This latter pattern appears to explain much of the variation observed, given that different spore production strategies appear to result in similar social fitness as a result of trade-offs. Under this scenario, different spore production strategies are nearly neutral in terms of their influence on social fitness (Figure 3), and hence the continuum of social behavioral strategies seen in these genotypes may simply reflect low selection pressure on social traits.

It is important to note, however, that although we have shown that relative representation in the sporehead is a poor measure of true social success, when interactions are viewed from the perspective of realized social fitness (which includes both spore number and viability) we find that there remains variation in social fitness that should reflect the true nature of cheaters and losers in this system (Figure 3). Similarly, in other microbial systems such as *Myxococcus* and *Pseudomonas*, cheater genotypes that exploitatively outcompete cooperators in mixed groups have been described when social fitness is measured in terms of the relative production of viable spores or cells, respectively [5, 10]. This is almost certainly due to the fact that microbes have complex life cycles and live in heterogeneously structured environments where diverse intra- and interspecific dynamics will interact to affect fitness. Other life history traits that we have not examined are no doubt manifest

in these ecologically relevant scenarios, and these in turn may directly or indirectly influence the coexistence of apparent social traits [36].

Our study therefore has clear implications for understanding the evolution of social traits in terms of cheater or cooperator strategies. Most notably, our results illustrate the importance of considering life history trade-offs when assessing social fitness: although many social systems, such as *D. discoideum*, may appear unbalanced with individuals that appear to "win," these individuals are really no better off in terms of fitness than individuals that appear to "lose." These observations may thus explain the paradoxical coexistence of substantial genetic variation in apparent social success in this and potentially other social organisms.

SUPPLEMENTAL INFORMATION

Supplemental Information includes one figure, one table, and Supplemental Experimental Procedures and can be found with this article online at http://dx.doi.org/10.1016/j.cub.2015.02.061.

AUTHOR CONTRIBUTIONS

C.R.L.T., D.R., and J.B.W. conceived and designed the project and wrote the manuscript. J.A.H. performed the measurements of social success and spore numbers. K.P. and N.G. performed the measurements of spore size and viability. D.M. and J.B.W. designed the data analyses. D.M. performed all analyses.

ACKNOWLEDGMENTS

We thank Daizaburo Shizuka for assistance with the analysis of social dominance. This work was supported by funding from the Natural Environment Research Council (UK) to C.R.L.T., D.R., and J.B.W.; the Biotechnology and Biological Research Council to J.B.W.; a Wellcome Trust Investigator Award to C.R.L.T.; and FAPESP grant numbers 2011/14295-7 and 2014/01694-9 and a Visiting Postgraduate Scholar grant from the University of Bath to D.M.

Received: November 5, 2014
Revised: January 20, 2015
Accepted: February 20, 2015
Published: March 26, 2015

REFERENCES

1. Strassmann, J.E., Queller, D.C., Avise, J.C., and Ayala, F.J., eds. (2012). In In the Light of Evolution, *Volume V: Cooperation and Conflict* (The National Academies Press).
2. Sachs, J.L., Mueller, U.G., Wilcox, T.P., and Bull, J.J. (2004). The evolution of cooperation. Q. Rev. Biol. *79*, 135–160.
3. Ghoul, M., Griffin, A.S., and West, S.A. (2014). Toward an evolutionary definition of cheating. Evolution *68*, 318–331.
4. Popat, R., Crusz, S.A., Messina, M., Williams, P., West, S.A., and Diggle, S.P. (2012). Quorum-sensing and cheating in bacterial biofilms. Proc. Biol. Sci. *279*, 4765–4771.
5. Velicer, G.J., Kroos, L., and Lenski, R.E. (2000). Developmental cheating in the social bacterium *Myxococcus xanthus*. Nature *404*, 598–601.
6. Zhang, Q.G., Buckling, A., Ellis, R.J., and Godfray, H.C. (2009). Coevolution between cooperators and cheats in a microbial system. Evolution *63*, 2248–2256.
7. MacLean, R.C., Fuentes-Hernandez, A., Greig, D., Hurst, L.D., and Gudelj, I. (2010). A mixture of "cheats" and "co-operators" can enable maximal group benefit. PLoS Biol. *8*, e1000486.
8. West, S.A., Griffin, A.S., Gardner, A., and Diggle, S.P. (2006). Social evolution theory for microorganisms. Nat. Rev. Microbiol. *4*, 597–607.
9. West, S.A., Diggle, S.P., Buckling, A., Gardner, A., and Griffin, A.S. (2007). The social lives of microbes. Annu. Rev. Ecol. Evol. Syst. *38*, 53–77.
10. Sandoz, K.M., Mitzimberg, S.M., and Schuster, M. (2007). Social cheating in *Pseudomonas aeruginosa* quorum sensing. Proc. Natl. Acad. Sci. USA *104*, 15876–15881.
11. Vos, M., and Velicer, G.J. (2009). Social conflict in centimeter-and global-scale populations of the bacterium *Myxococcus xanthus*. Curr. Biol. *19*, 1763–1767.
12. Strassmann, J.E., Zhu, Y., and Queller, D.C. (2000). Altruism and social cheating in the social amoeba Dictyostelium discoideum. Nature *408*, 965–967.
13. Li, S.I., and Purugganan, M.D. (2011). The cooperative amoeba: *Dictyostelium* as a model for social evolution. Trends Genet. *27*, 48–54.
14. Shaulsky, G., and Kessin, R.H. (2007). The cold war of the social amoebae. Curr. Biol. *17*, R684–R692.
15. Strassmann, J.E., and Queller, D.C. (2011). Evolution of cooperation and control of cheating in a social microbe. Proc. Natl. Acad. Sci. USA *108* (2), 10855–10862.
16. Gilbert, O.M., Foster, K.R., Mehdiabadi, N.J., Strassmann, J.E., and Queller, D.C. (2007). High relatedness maintains multicellular cooperation in a social amoeba by controlling cheater mutants. Proc. Natl. Acad. Sci. USA *104*, 8913–8917.
17. Santorelli, L.A., Thompson, C.R., Villegas, E., Svetz, J., Dinh, C., Parikh, A., Sucgang, R., Kuspa, A., Strassmann, J.E., Queller, D.C., and Shaulsky, G. (2008). Facultative cheater mutants reveal the genetic complexity of cooperation in social amoebae. Nature *451*, 1107–1110.
18. Khare, A., Santorelli, L.A., Strassmann, J.E., Queller, D.C., Kuspa, A., and Shaulsky, G. (2009). Cheater-resistance is not futile. Nature *461*, 980–982.
19. Santorelli, L.A., Kuspa, A., Shaulsky, G., Queller, D.C., and Strassmann, J.E. (2013). A new social gene in *Dictyostelium discoideum, chtB*. BMC Evol. Biol. *13*, 4.
20. Fortunato, A., Queller, D.C., and Strassmann, J.E. (2003). A linear dominance hierarchy among clones in chimeras of the social amoeba Dictyostelium discoideum. J. Evol. Biol. *16*, 438–445.
21. Buttery, N.J., Thompson, C.R., and Wolf, J.B. (2010). Complex genotype interactions influence social fitness during the developmental phase of the social amoeba Dictyostelium discoideum. J. Evol. Biol. *23*, 1664–1671.
22. Buttery, N.J., Rozen, D.E., Wolf, J.B., and Thompson, C.R. (2009). Quantification of social behavior in D. discoideum reveals complex fixed and facultative strategies. Curr. Biol. *19*, 1373–1377.
23. Francis, D., and Eisenberg, R. (1993). Genetic structure of a natural population of Dictyostelium discoideum, a cellular slime mould. Mol. Ecol. *2*, 385–391.
24. Shizuka, D., and McDonald, D.B. (2012). A social network perspective on measurements of dominance hierarchies. Anim. Behav. *83*, 925–934.
25. Van Dyken, J.D., Linksvayer, T.A., and Wade, M.J. (2011). Kin selection-mutation balance: a model for the origin, maintenance, and consequences of social cheating. Am. Nat. *177*, 288–300.
26. Roff, D.A. (1992). The Evolution of Life Histories: Theory and Analyses. (Chapman & Hall).
27. Stearns, S. (1992). The Evolution of Life Histories. (Oxford University Press).
28. Morris, D.W. (1987). Optimal allocation of parental investment. Oikos *49*, 332–339.
29. Parker, G.A., and Begon, M. (1986). Optimal egg size and clutch size: effects on environment and maternal phenotype. Am. Nat. *128*, 573–592.
30. Hadfield, J.D. (2010). MCMC methods for multi-response generalized linear mixed models: the MCMCglmm R package. J. Stat. Softw. *33*, 1–22. http://www.jstatsoft.org/v33/i02/paper.
31. Zhang, X.-X., and Rainey, P.B. (2013). Exploring the sociobiology of pyoverdin-producing *Pseudomonas*. Evolution *67*, 3161–3174.

32. Walsh, B., and Blows, M.W. (2009). Abundant genetic variation + strong selection = multivariate genetic constraints: a geometric view of adaptation. Annu. Rev. Ecol. Evol. Syst. *40*, 41–59.

33. Parkinson, K., Buttery, N.J., Wolf, J.B., and Thompson, C.R. (2011). A simple mechanism for complex social behavior. PLoS Biol. *9*, e1001039.

34. Muramoto, T., and Chubb, J.R. (2008). Live imaging of the *Dictyostelium* cell cycle reveals widespread S phase during development, a G2 bias in spore differentiation and a premitotic checkpoint. Development *135*, 1647–1657.

35. Zimmerman, W., and Weijer, C.J. (1993). Analysis of cell cycle progression during the development of *Dictyostelium* and its relationship to differentiation. Dev. Biol. *160*, 178–185.

36. Sathe, S., Khetan, N., and Nanjundiah, V. (2014). Interspecies and intraspecies interactions in social amoebae. J. Evol. Biol. *27*, 349–362.

Current Biology
Supplemental Information

Fitness Trade-offs Result in the Illusion of Social Success

Jason B. Wolf, Jennifer A. Howie, Katie Parkinson, Nicole Gruenheit, Diogo Melo, Daniel Rozen, and Christopher R.L. Thompson

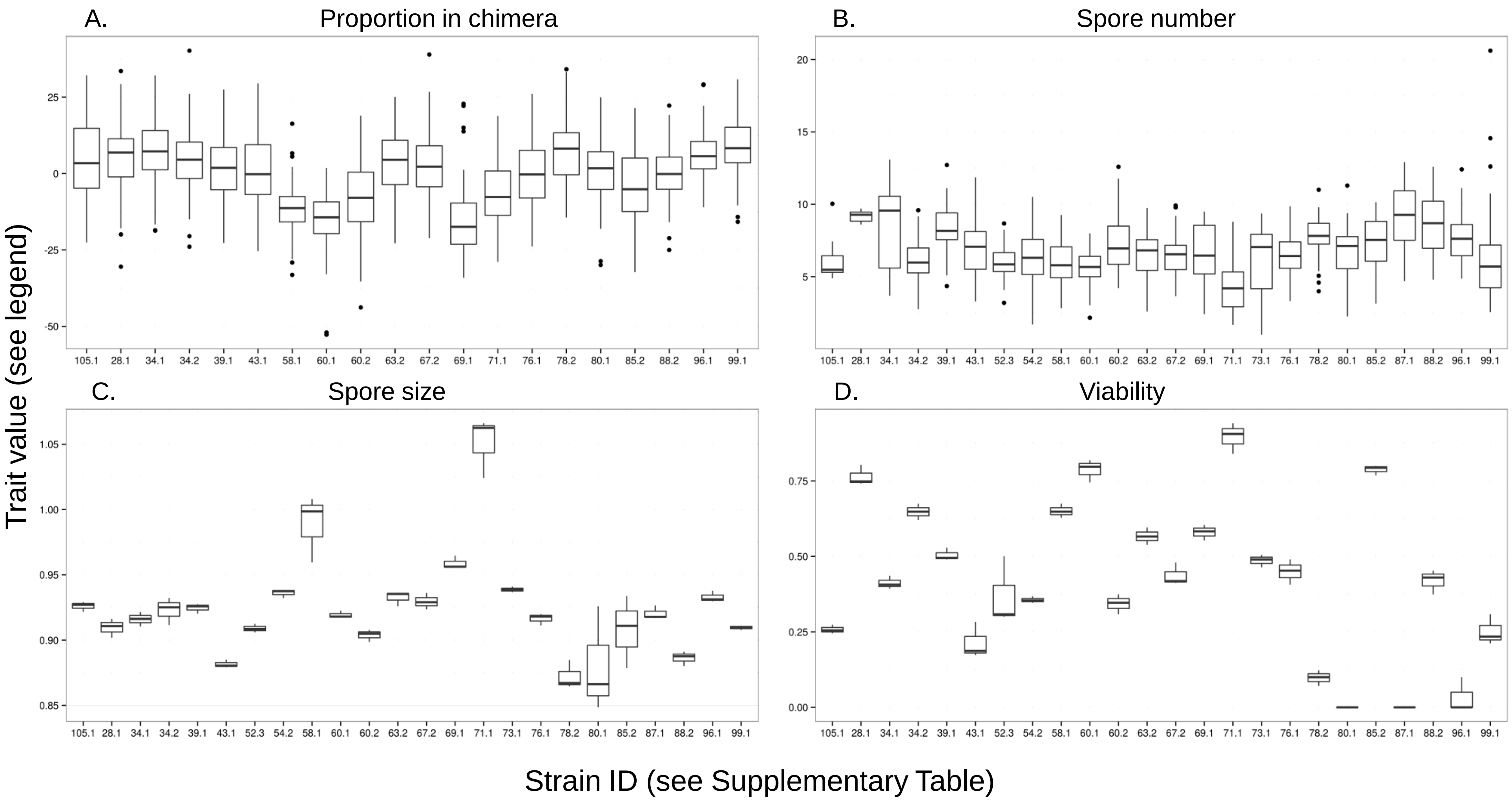
A.
Proportion in chimera
B.
Spore number
C.
Spore size
D.
Viability
Trait value (see legend)
Strain ID (see Supplementary Table)
105.1
28.1
34.1
34.2
39.1
43.1
52.3
54.2
58.1
60.1
60.2
63.2
67.2
69.1
71.1
73.1
76.1
78.2
80.1
85.2
87.1
88.2
96.1
99.1

Supplementary Figure 1

The distribution of the four traits for each strain. For each trait, strains are illustrated using a standard box plot, where the box extends from the first to the third quartiles (the 25th and 75th percentiles) with a line within the box indicating the location of the median. The upper and lower lines (whiskers) extend from the box to the highest and lowest values that are within 1.5 times the inter-quartile range (i.e., distance between the first and third quartiles). Data beyond the end of the whiskers are outliers and appear as individual points. A. The distribution of proportional representation in chimeric fruiting bodies. Values shown have been corrected for the effect of labelling and the social partner (to account for unbalanced combinations). The values are scaled such that they show the deviation expected from 50:50, where a value of zero means that the expected representation in chimeric sporeheads is 50%, while positive values show the expected degree of overrepresentation and negative values show the expected degree of underrepresentation. For example, a value of 25 indicates that the strain has an average representation in chimeric sporeheads of 75%. B. The number of spores in a clonal sporehead in units of 10^5 spores (e.g., a value of 8 would indicate 8 x 10^5 spores). The values shown have been adjusted to account for experimental block effects. C. The size of spores. Values plotted are the $\log_{10}$ of the raw values in μm, which is the scale used in the analyses (see Methods for more details). D. The viability of spores as a percentage. Values plotted are the squaren root of the raw values, which is the scale used in the analyses (see Methods for more details). For relationships between these data see Figure 1.

Supplementary Table 1

Strains (genotypes) used in all studies. All strains were originally isolated from Little Butt's Gap, North Carolina, USA [1] and obtained from the Dicty Stock Center. Strains listed in red were not used in the assays for social success. For trait data associated with these strains, see Figure 1.

NC28.1	NC43.1	NC60.1	NC69.1	NC78.2	NC88.2
NC34.1	NC52.3	NC60.2	NC71.1	NC80.1	NC96.1
NC34.2	NC54.2	NC63.2	NC73.1	NC85.2	NC99.1
NC39.1	NC58.1	NC67.2	NC76.1	NC87.1	NC105.1

EXPERIMENTAL PROCEDURES

Growth and maintenance of genotypes

Naturally occurring genotypes of *D. discoideum* used throughout the study were isolated from the same geographic region of Little Butt's Gap, North Carolina [1]. All traits were measured on a set of 24 strains (see Supplementary Table 1 for a list of strain IDs), except social success, which was measured for a subset of 20 strains. Genotypes were acquired from the Dicty Stock Centre and subsequently stored as frozen stocks. For experiments, stocks were grown on Schaeffer's sporulation medium (SM) agar plates in association with *Klebsiella aerogenes* (Ka) bacteria. For amoeba growth, 'clearing plates' containing 5 x 10^5 cells were plated with Ka and incubated at 22°C for approximately 36 hours. Growing cells were harvested before they began to aggregate and bacteria were removed by repeated washing and differential centrifugation in KK2 (16.1 mM KH_2PO_4, 3.7mM K_2HPO_4).

Estimating social success in chimera

Growing cells of each genotype were harvested, and re-suspended at 1 x 10^7 cells/ml. For fluorescent labelling, cells were shaken for 30 minutes with 10mM CMFDA Cell Tracker Green dissolved in DMSO; control cells were shaken with DMSO. For development, cells were plated on 1.5% KK2 L28 purified agar plates at a final density of 1.6 x 10^6 cells per cm^2. Different genotypes were mixed in a 50:50 ratio. The social success of each genotype was estimated by counting the percentage of fluorescent and non-fluorescent spores using a CyAn flow cytometer. Estimates of social success were made using all possible reciprocal pair-wise combinations of 20 genotypes. Each of the 380 pair-wise combinations was replicated an average of 5.16 times, resulting in a total sample size of 1960 measures of social success. Social success estimates were averaged across social partners and corrected for the labelling effect, using a mixed modelling approach. We modelled the fixed effect of Cell Tracker labelling and the random effect of the partner genotype on the proportion of the genotype in a chimeric fruiting body using the Mixed Procedure in SAS version 9.4 (SAS Institute, Cary, NC, USA), fitted by restricted maximum likelihood (REML). The residuals from this model were used for subsequent analyses.

To determine the degree of linearity (transitivity) in the pair-wise social success measures, we used the network measure 'triangle transitivity (t_{tri})' developed in Shizuka and McDonald [2], which tests the proportion of triads that are transitive relative to those that are non-transitive, scaled to the null expectation. The results are essentially identical using other

standard measures such as Kendall's *K* and Landau's *h* [see 2 for further details], so only a single measure is reported.

Spore size and viability

For estimates of spore size, strains were hatched onto SM agar with Ka bacteria and spores harvested from the resulting fruiting bodies into spore buffer (20mM EDTA and 0.1% NP-40). Spores were imaged at 40x magnification. Automated measurements of spore size (length, width, and total area) were made from three images per plate using ImageJ software [3]. Spores of each genotype were measured in three independent replicates, with at least 77 spores measured from each strain in each block (with a mean of 228.7 spores per strain per block for a total of 16,464 spores measured overall). Length, width and total area measures of spore size are highly intercorrelated, so we used spore length as the best estimate of spore size because it has the highest repeatability (in terms of the proportion of variance among strains). We used the replicated measures within each block to generate three completely independent estimates of spore size for each strain (i.e., one measure per strain per block). Because spore size is not normally distributed, we used the median spore size in each block as the best measure of spore size (almost identical results are achieved using the mean spore size in each replicate). The median spore sizes were then $\log_{10}$ transformed to achieve a normally distributed measure of spore size. This measure of spore size has a strain level repeatability of 71%. To measure spore viability, spores were harvested into spore buffer, counted using a haemocytometer and diluted to a density of $2x10^3$ cells/ml. 200 spores were plated on SM agar with Ka bacteria with three replicates per strain. The number of visible clear plaques was counted after 4 days growth at 22 deg. To measure spore size and viability after chimeric development in 50:50 mixes, one genotype was labelled with CMFDA Cell Tracker as described above. Fluorescent and non-fluorescent spores were separated using a FACS Aria cell sorter, before size and viability were determined using the above methods. We found significant correlations between chimeric and clonal spore size (r = 0.94, p = 0.008) and spore viability (r = 0.84, p = 0.039), in a subset strains tested in pair-wise mixes. Therefore, clonal trait measures provide good estimates of chimeric traits, allowing us to estimate socially relevant traits through clonal development.

Spore number

To estimate total clonal spore counts, all fruiting bodies were washed from a plate containing clonal fruiting bodies into a known volume of spore buffer and the total number of spores

was estimated by direct haemocytometer counts. Each genotype was measured in at least 8 independent replicates, with an average of 33.6 replicates per strain. To account for differences across experimental blocks, we modelled the random effect of block on the spore count using the Mixed Procedure in SAS version 9.4 (SAS Institute, Cary, NC, USA), fitted by restricted maximum likelihood (REML). The residuals from this model were used for subsequent analyses.

Quantitative genetic analyses

Proportions of among strain variation in traits, which can be interpreted as either repeatabilities of a trait at the strain level of as broad-sense heritabilities (H^2), were estimated from the proportion of variance attributed to the random effect of genotype in a mixed model, fitted using restricted maximum likelihood (REML) in the Mixed Procedure in SAS version 9.4 (SAS Institute, Cary, NC, USA). Significance was estimated using a likelihood ratio test.

To estimate the genetic correlation between traits we first standardized each trait to a mean of zero and a variance of one. The four traits were used in a multivariate model fitted using MCMCglmm [4] using a Bayesian modification of the framework described by Fry [5] to estimate genetic variances of and genetic correlations among the four traits measured in the set of genotypes. In this model, the four traits are treated as measures of the same underlying trait and the genotype is used as the unit of repeated measurements. The model estimates the correlations for the traits at the level of genotypes, which represent genetic correlations since the traits were measured independently [see 5 for further details]. We used weakly informative independent Gaussian priors for the residual and mixed effect (genetic) variances. Model convergence was accessed by inspection of variable traces. The genetic correlations between traits were estimated as the mean of the posterior distribution. This estimate was similar to results from a maximum likelihood fitted model using SAS or lme4, with the added advantage that the posterior distribution of covariances can be used to create high probability confidence intervals (CI) for all heritabilities and genetic correlations, taking into account all sources of uncertainty in the system and allowing a straight forward test for significant difference from zero for all correlations [6]. To visualize the relationships between traits we used the posterior sampled covariance matrices and means to create a data set of simulated strains by drawing values from normal distributions in the scaled trait space. These values were plotted along with means for each observed strain to demonstrate the agreement between data and posterior simulations. These visualizations from the simulations also

illustrate the uncertainty for each of the relations between traits, and again their distribution takes into account every source of underlying uncertainty.

Modelling fitness

To understand the nature of selection on traits we first estimated the means and variances of the unscaled traits using MCMCglmm and, along with the correlations estimated for the scaled traits, used them to generate posterior simulations of strains in the unscaled space. We calculated expected 'social fitness' as the product of social success and spore viability (so relative representation in the clonal fruiting body discounted by the viability of the spores produced). To calculate fitness in each of the simulated strains we first transformed viability (which was estimated using the residuals from the mixed model) to the [0, 1] interval via an inverse logit transformation and scaled success to the same interval (so social success represents the average relative representation in the sporehead for a given genotype). Social fitness, as the product between viability and success, is then restricted to values between zero and one.

To understand the relationship between the underlying life-history traits (spore number and spore size) and components of fitness, we estimated the linear relationship between these traits and each of the fitness components (social success and viability). The net relationship between these traits and total fitness (i.e., the overall shape of the selection surface) was visualized using a quadratic regression curve.

References

S1. Francis, D., and Eisenberg, R. (1993). Genetic structure of a natural population of Dictyostelium discoideum, a cellular slime mold. Mol. Ecol. *2*, 385-392.

S2. Shizuka, D., and McDonald, D.B. (2012). A social network perspective on measurements of dominance hierarchies. Animal Behaviour *83*, 925-934.

S3. Schneider, C.A., Rasband, W.S., and Eliceiri, K.W. (2012). NIH Image to ImageJ: 25 years of image analysis. Nature Methods *9*, 671-675.

S4. Hadfield, J.D. (2010). MCMC Methods for Multi-Response Generalized Linear Mixed Models: The MCMCglmm R Package. Journal of Statistical Software *33*, 1-22.

S5. Fry, J.D. (2004). Estimation of genetic variances and covariances by restricted maximum likelihood using Proc Mixed. In Genetic analysis of complex traits using SAS, A.M. Saxton, ed. (Cary, N.C.: SAS Institute), pp. 11-34.

S6. Gelman, A., Carlin, J.B., Stern, H.S., Vehtari, A., and Rubin, D.B. (2013). Bayesian Data Analysis, Third Edition, (Chapman & Hall).
